# Supplementary material for: Global Analysis of the WOX Transcription Factor Family in Akebia trifoliata
Source: Curr Issues Mol Biol. 2023 Dec 19;46(1):11–24. doi: 10.3390/cimb46010002 (PMC10814775; doi:10.3390/cimb46010002)
Supplement: Supplementary file 1 [file cimb-46-00002-s001.zip › Supplementary Figure S1-S4.pdf]

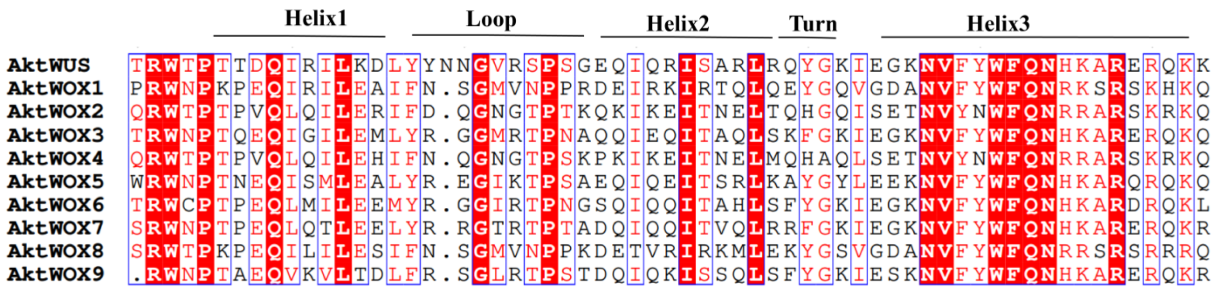

Figure S1 Alignment of the homeodomain sequences. Reds indicate residues that are highly conserved in homeodomains. The red part indicates high homology.

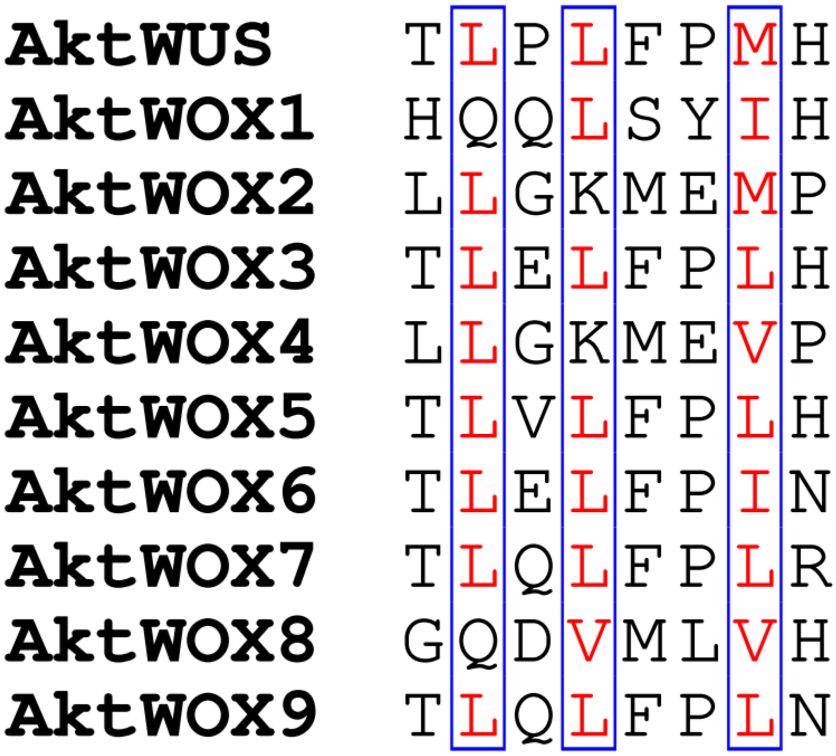

Figure S2 Alignment of the WUS box that is located downstream of the homeodomain.The red part indicates high homology.

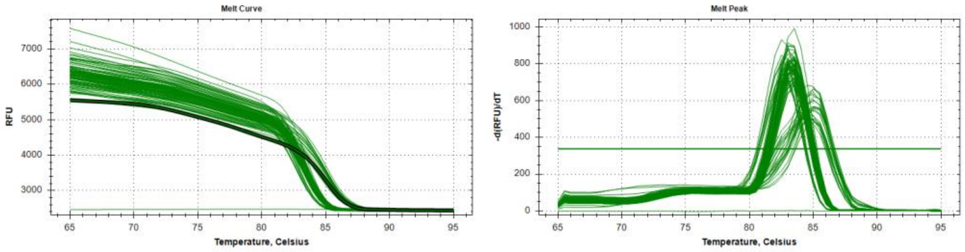

Figure S3 Amplification and dissolution curves of qRT-PCR

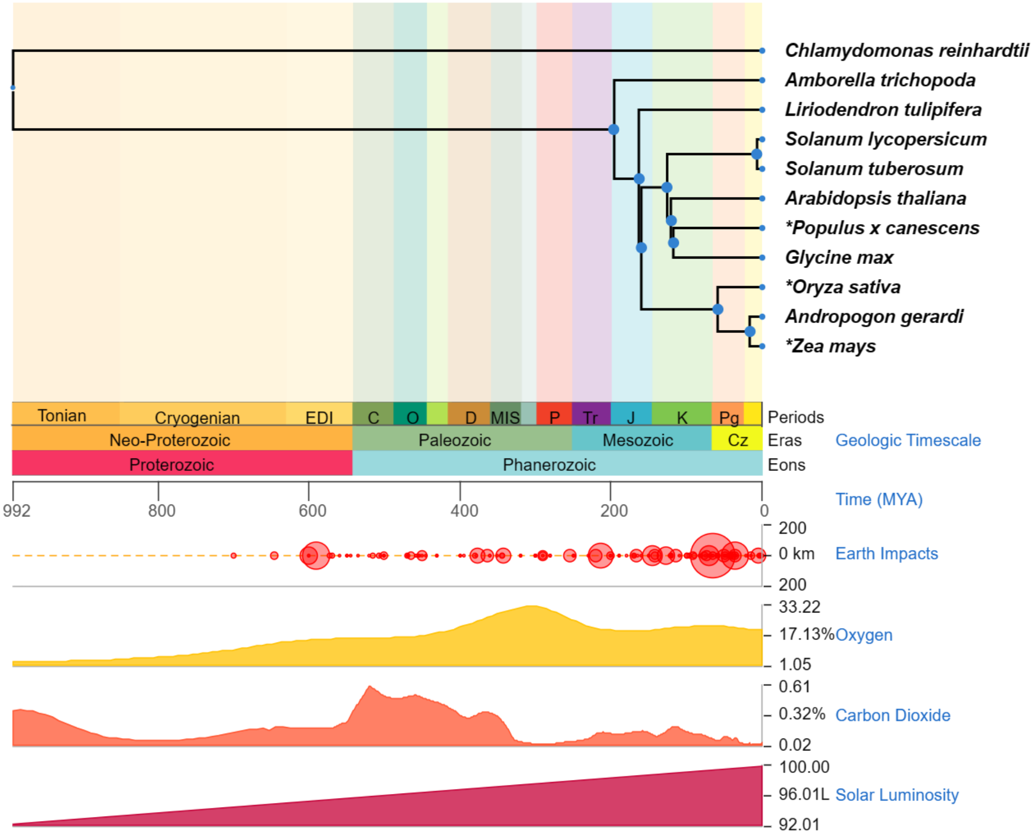

Figure S4 The evolution of twelve species over time. "MYA" represent millions of years ago, The divergence time of *Akebia trifoliata* and other 11 species by the "Get Divergence Time" search function. The median time and its confidence interval are derived from the TToL5 database. Solar luminosity (furthest left, red), global CO_2 levels (second from left, orange), O_2 levels (thirds from left, yellow), and major earth impact events (circles) are also shown. * indicate divergences without any names in the NCBI database.
